# Tissue-resident memory T cells in the era of (Neo) adjuvant melanoma management

**DOI:** 10.3389/fimmu.2022.1048758

**Published:** 2022-11-16

**Authors:** Kai R. Plunkett, Jesse D. Armitage, Andrisha-Jade Inderjeeth, Alison M. McDonnell, Jason Waithman, Peter K. H. Lau

**Affiliations:** ^1^ School of Biomedical Sciences, University of Western Australia, Nedlands, WA, Australia; ^2^ Telethon Kids Institute, University of Western Australia, Nedlands, WA, Australia; ^3^ Department of Medical Oncology, Peter MacCallum Cancer Centre, Melbourne, VIC, Australia; ^4^ Melanoma Discovery Laboratory, Harry Perkins Institute of Medical Research, Nedlands, WA, Australia; ^5^ Department of Medical Oncology, Sir Charles Gairdner Hospital, Nedlands, WA, Australia

**Keywords:** tissue-resident memory CD8(+) T cells, cancer immunotherapy, melanoma, immune checkpoints, neoadjuvant immunotherapy, adjuvant immunotherapy, tumor microenvironment

## Abstract

Tissue-resident memory T (T_RM_) cells have emerged as key players in the immune control of melanoma. These specialized cells are identified by expression of tissue retention markers such as CD69, CD103 and CD49a with downregulation of egress molecules such as Sphingosine-1-Phosphate Receptor-1 (S1PR1) and the lymphoid homing receptor, CD62L. T_RM_ have been shown to be integral in controlling infections such as herpes simplex virus (HSV), lymphocytic choriomeningitis virus (LCMV) and influenza. More recently, robust pre-clinical models have also demonstrated T_RM_ are able to maintain melanoma in a dormant state without progression to macroscopic disease reminiscent of their ability to control viral infections. The discovery of the role these cells play in anti-melanoma immunity has coincided with the advent of immune checkpoint inhibitor (ICI) therapy which has revolutionized the treatment of cancers. ICIs that target programmed death protein-1 (PD-1) and cytotoxic T lymphocyte antigen-4 (CTLA-4) have led to substantial improvements in outcomes for patients with metastatic melanoma and have been rapidly employed to reduce recurrences in the resected stage III setting. While ICIs mediate anti-tumor activity *via* CD8^+^ T cells, the specific subsets that facilitate this response is unclear. T_RM_ invariably exhibit high expression of immune checkpoints such as PD-1, CTLA-4 and lymphocyte activating gene-3 (LAG-3) which strongly implicates this CD8^+^ T cell subset as a crucial mediator of ICI activity. In this review, we present pre-clinical and translational studies that highlight the critical role of T_RM_ in both immune control of primary melanoma and as a key CD8^+^ T cell subset that mediates anti-tumor activity of ICIs for the treatment of melanoma.

## Introduction

Immune checkpoint inhibitors that target the PD-1 axis form the backbone of systemic treatment for cutaneous malignancies, namely melanoma (1), cutaneous squamous cell carcinoma (cSCC) (2) and Merkel cell carcinoma (3). Original indications were approved in metastatic melanoma, leading to unprecedented long-term survival outcomes with 5-year overall survival (OS) rates exceeding 50% with combination ipilimumab-nivolumab (anti-CTLA-4 and anti-PD-1) (4). Given these substantial improvements in outcomes for patients with metastatic disease, ICIs were quickly investigated in the resected stage III melanoma setting where adjuvant anti-PD-1 with pembrolizumab was shown to reduce recurrence by approximately 40% compared to placebo (5). Recently, several studies display encouraging activity of ICI in the neoadjuvant preoperative setting, which promises to be a new treatment paradigm for the management of stage III melanoma (6–8).

Given the shift towards adjuvant and neoadjuvant immunotherapy for the treatment of melanoma, a critical understanding of the tumor microenvironment (TME) within the skin is required to inform new therapeutic combinations with anti-PD-1. Notably, a pre-requisite for anti-tumor responses to ICI include the presence of T cells co-located at the tumor margin (9). However, an important sub-class of T cells, tissue-resident memory CD8^+^ T (T_RM_) cells are major players in mediating anti-tumor responses to ICI (10, 11). T_RM_ permanently reside in tissues and are unable to circulate due to the lack of lymphoid homing markers CCR7 and CD62L. Additionally, skin T_RM_ are identified by the increased expression of the C-type lectin receptor CD69 and the αE subunit of the αEβ7 integrin CD103 (12–14). Critically, tumor-infiltrating T_RM_ express immune checkpoint molecules including PD-1, CTLA-4, and LAG-3. T_RM_ are also a rich source of type I interferons (IFN), IFN-γ and tumor necrosis factor alpha (TNF-α) which are essential for anti-tumor activity (11, 15).

T_RM_ were originally described as having central roles in immune control of viral infections such as HSV and LCMV (13, 16, 17). Moreover there is a substantial body of evidence that indicates a similar critical role for T_RM_ in both immune control of melanoma and mediating responses to ICIs (12). Seminal pre-clinical models show T_RM_ are vital in controlling cutaneous melanoma by inducing a state of immune-mediated equilibrium which is reminiscent of HSV dormancy where the host immune system suppresses but does not completely eliminate the virus (12, 13). In humans, the presence and abundance of T_RM_ is associated with improved prognosis in patients with melanoma, T_RM_ isolated from human melanoma specimens display high PD-1 and LAG-3 expression corroborating pre-clinical models (10, 18). This finding has critical therapeutic importance given the recent successful development of combination anti-LAG-3 with anti-PD-1 in metastatic melanoma (19). Moreover, tumor-infiltrating T_RM_ correlate with longer disease-free periods and OS in patients of cSCC (20, 21), breast (22), ovarian (23), endometrial (24), pancreatic ductal adenocarcinoma (25) and lung cancer (26, 27) which further exemplifies the importance of this T cell subset in cancer outcomes.

With neoadjuvant trials and the recent success of adjuvant treatment in melanoma, the stage is set for a new raft of immunotherapy studies to further improve patient outcomes. The past 10 years has produced a stunning paradigm shift in the treatment of skin malignancies but the precise immunological mechanisms that underpin ICI-driven anti-tumor responses remain unclear. This review will explore the basic biology of T_RM_ and their role in mediating clinical responses to ICI in cutaneous malignancies.

## Hallmarks of tissue-resident memory T cells

### Phenotypic features

Over the past two decades our understanding of CD8 memory T cell subsets have evolved considerably. Stem memory T cells (T_SCM_, defined by CD45RA^+^CCR7^+^CD27^+^CD95^+^) (28) represent the least differentiated memory subset and display a multipotency potential to differentiate into two major memory populations central memory (T_CM_) and effector memory (T_EM_) cells (29, 30). T_CM_ primarily reside in lymphoid organs and are reactivated following antigen stimulation, such as during secondary viral infections. Expression of the chemokine receptor CCR7 and the lymphoid specific L-selectin CD62L allow T_CM_ to migrate from the lymph or blood vessels to enter secondary lymphoid organs (31). In contrast, T_EM_ are memory cells that do not express CCR7 and CD62L, but display chemokine receptors, such as CXCR3 and adhesion molecules, such as intracellular adhesion molecule 1 (ICAM-1), that allow entry into inflamed tissues (32). T_EM_ exert cytotoxic activities on target cells *via* their ability to release IFN-γ, granzymes and perforins. Moreover, terminally differentiated effector memory (T_EMRA_) represent a highly differentiated population of memory CD45RA^+^ T cells with low CD62L and CCR7 low expression alongside high cytolytic capability (30). Both T_CM_ and T_EM_ form the group of circulating memory T (T_CIRC_) cells as they circulate *via* the lymphatics and blood stream (33).

More recently, a third major population of CD8^+^ memory T cells, T_RM_ have been identified. T_RM_ permanently reside in tissue following infection or malignancy and do not re-enter circulation following establishment of a population in the site (12). Originally described in viral models, T_RM_ arise from killer cell lectin-like receptor G1 (KLRG1) negative population of memory precursor cells, which lack the lymphoid homing receptor CD62L and the tissue egress receptor S1PR1 (34). S1PR1 and its ligand, sphinosine-1-phosphate (S1P) is a critical signaling pathway for the egress of effector CD8^+^ T cells from lymphoid organs to sites of infection (35). Until recently it was unclear which type of memory T cells are progenitors for T_RM_ in the skin. However work by Matos et al. indicates T_CM_ display higher skin tropism than T_EM_, and are therefore more efficient progenitors for skin T_RM_ (36).

**Figure 1:**
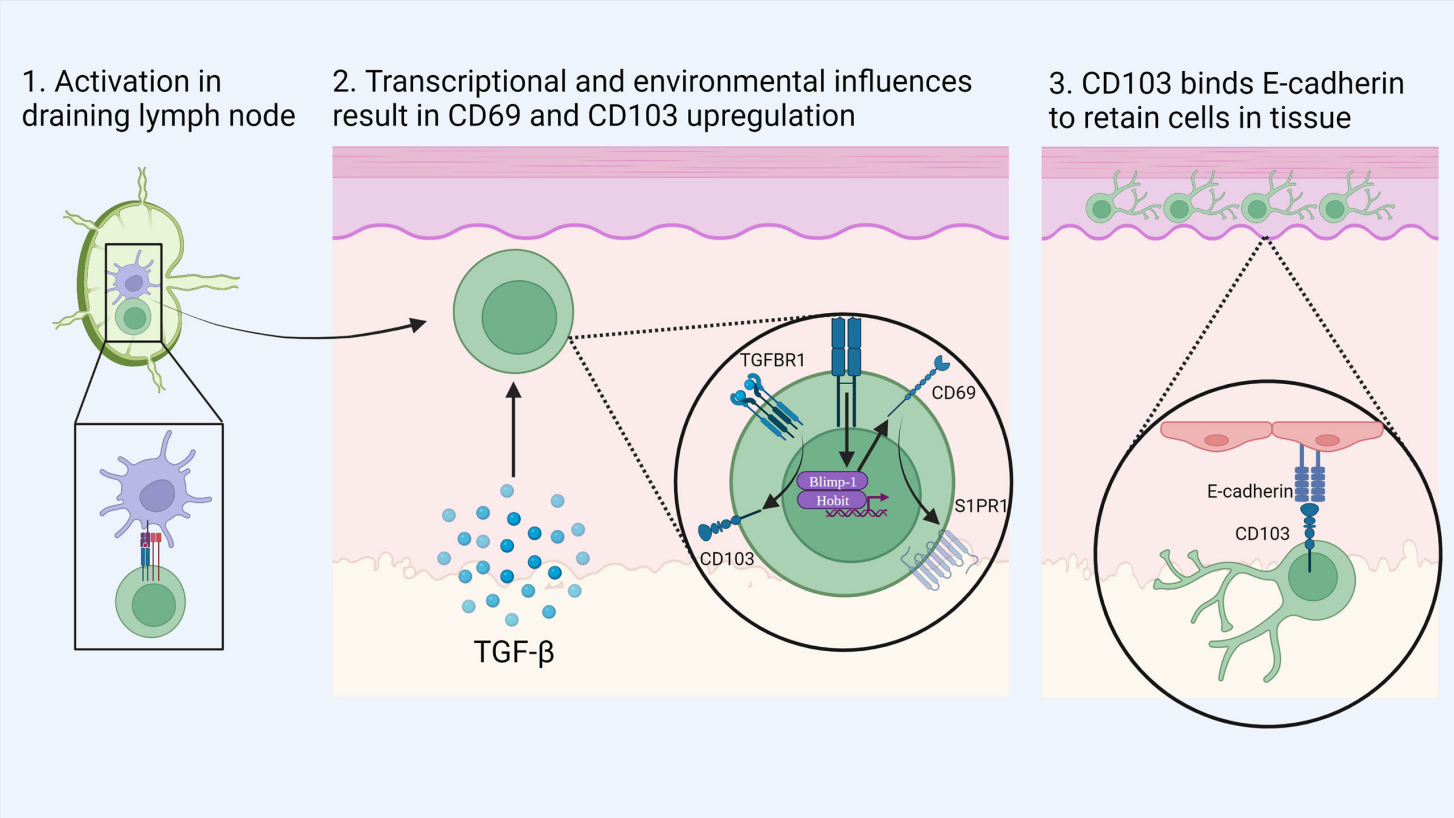
Development of skin T_RM_: Naïve CD8^+^ T cells are activated by dendritic cells in the local draining lymph node. Upon migration to the skin, local environmental factors such as TGF-β induce the transition to a T_RM_ phenotype. Activation of the transcription factors Blimp-1 and Hobit drives the expression of CD69 which in turn causes the down regulation of S1PR1. In addition, TGF-β stimulates the upregulation of CD103 which binds to E-cadherin in the epidermis to retain cells in tissue. Created with Biorender.com.

**Figure 2:**
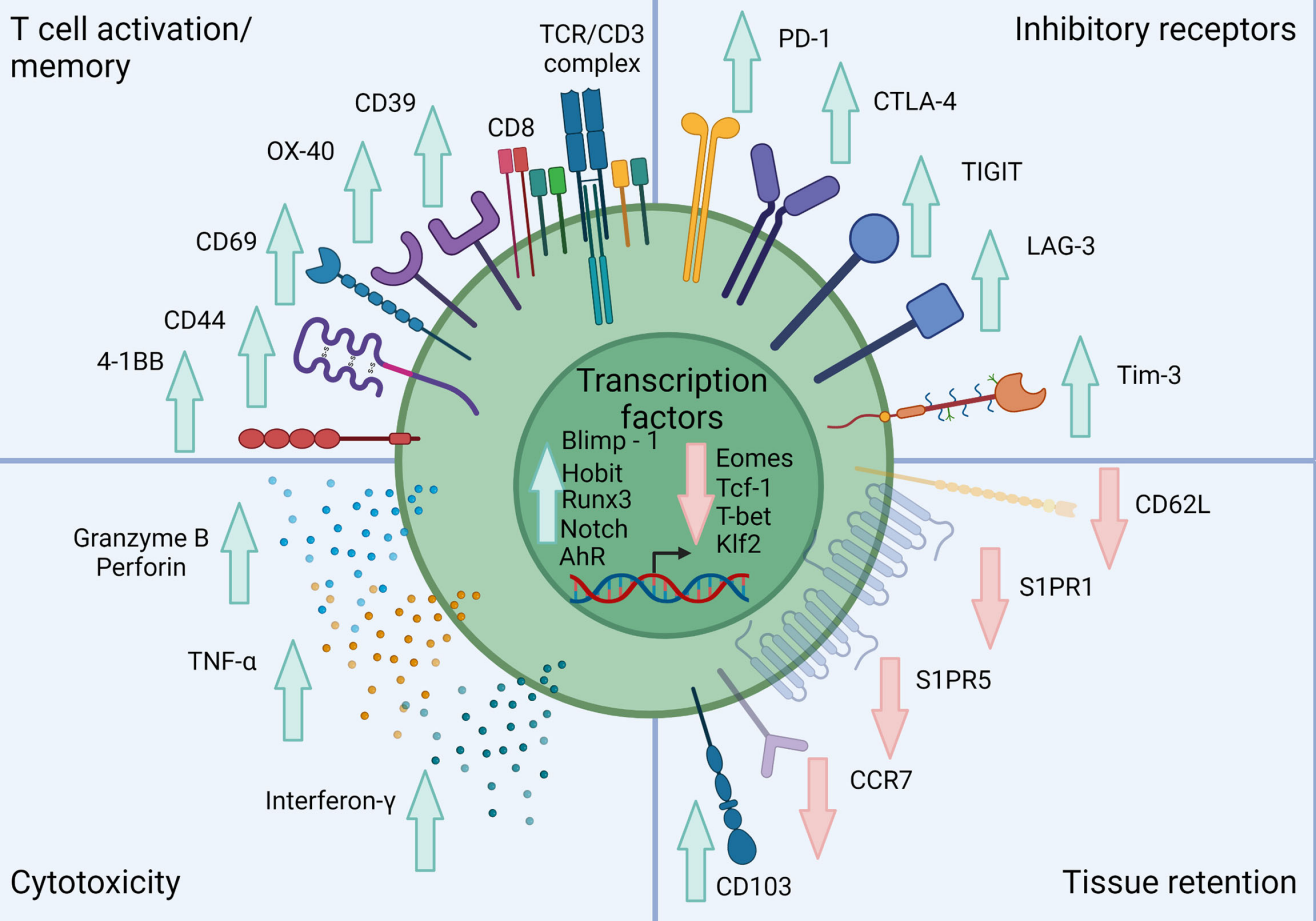
Classical phenotype of skin T_RM_. Skin T_RM_ up regulate markers of memory such as CD44, CD69 and CD103 while down-regulating the lymphoid homing molecule CCR7 and egress receptors CD62L, S1PR1 and S1PR5. Skin T_RM_ also express a range of inhibitory receptors including PD-1, CTLA-4 and LAG-3. These cells also exhibit a unique transcription factor profile exhibited by increased Blimp-1, Hobit, Runx, AhR and Notch expression with inhibition of Eomes, Tcf-1, T-bet and Klf2. They also show a conventional CD8^+^ T cell cytolytic profile including Granzyme B, Perforin, TNF-α and IFN-γ expression. Created with Biorender.com.

CD8^+^ T_RM_ can be distinguished from their circulating counterparts largely through expression of the key surface molecules CD69, CD103 and CD49a combined with reduced expression of tissue egress and lymph node homing markers such as S1PR1, CCR7, CD62L (17, 34, 37). CD103 associates with integrin β7 (CD49d) to form the αEβ7 integrin complex which binds the cell adhesion molecule E-cadherin and anchors T_RM_ within barrier tissues (38) (**Figure 1**). CD103 is most highly expressed by CD8^+^ T_RM_ in mucosal sites such as the lung and skin where its expression is driven by TGF-β. CD69 is a transmembrane C-type lectin receptor that is constitutively expressed on T_RM_ and contributes to the establishment of tissue residency *via* inhibition of S1PR1-mediated tissue egress. Loss of S1PR1 on CD8^+^ T cells leaves T cells unable to respond to S1P secreted by endothelial cells, maintaining their niche within tissues (38, 39) (**Figure 2**).

Several studies indicate that defining T_RM_ with CD69 and CD103 expression is tissue dependent (40). T_RM_ populations have been defined as CD69^+^ CD103^-^ in different anatomical sites such as intestines (41), secondary lymphoid organs (42), liver (40), kidney and adipose tissue (43). Although TGF-β signals up-regulation of CD103 expression by skin T_RM_, *Tgfbr2^-/-^* mouse models have shown liver T_RM_ can develop independent of TGF-β signaling (40). This indicates the unique immunological niche within each anatomical site plays a key role in the development of tissue-specific T_RM_.

CD49a is another crucial T_RM_ surface molecule and constitutes the α-subunit of the α1β1 integrin receptor, also known as very late antigen-1 (VLA-1). Similar to CD103, CD49a functions to maintain T_RM_ within tissues (44). In the skin, CD49a binds its cognate ligand collagen IV in the basement membrane which separates the dermis and epidermis to establish tissue residency (44). Both IL-12 and TGF-β can induce expression of CD49a but the former cytokine has been shown to inhibit CD103 expression *in vitro* (45). Expression of CD49a was found to delineate a functional subset of skin T_RM_ with CD8^+^CD49a^+^ producing IFN-γ whereas CD8^+^CD49^-^ T_RM_ produced IL-17 in psoriatic skin lesions (46). Furthermore CD49a also promotes dendritic extensions by skin T_RM_ which may enhance the cells antigen surveillance capability (45). CD49a expression on T_RM_ is correlated with improved outcomes in patients with melanoma (18).

While CD69, CD103 and CD49a have been used to discriminate T_RM_ from other memory subsets, CD39 has recently been described as a distinguishing marker of tumor specific CD8 TILs (26, 47). Using mass cytometry and T cell receptor (TCR) sequencing of colorectal and lung cancer specimens, CD39 expression was enriched in CD8 tumor infiltrating lymphocytes (TILs) that possessed cancer antigen specificity (47). In contrast CD39^-^ TIL exhibited T cell receptors to non-cancer epitopes including viral antigens such as cytomegalovirus or influenza virus. In a study by Duhen et al, all CD39^+^ cells also co-expressed both CD69 and CD103, representing a subset of tumor-reactive T_RM_ (26). Gene expression data showed CD39^+^ TILs exhibited much higher expression of immune checkpoints such as PD-1 and CTLA-4 compared to CD39^-^ TILs (26). Notably the CD39-CD73 axis has also been implicated with immunosuppressive roles (48, 49). CD39 metabolizes extracellular ATP and ADP to AMP, while CD73 converts AMP into adenosine (50). Extracellular adenosine signals through its cognate receptors, A_1_R, A_2A_R, A_2B_R and A_3_R and causes immunosuppression by proliferation of T_REG_ within the TME, increasing the release of anti-inflammatory cytokines such as IL-10 and TGF-β (51). Pre-clinical models have shown adenosine receptor antagonists can augment anti-tumor activity in combination with anti-PD-1 in pre-clinical models of melanoma (48), breast (48) and colon cancer (52). Although clinical trials of adenosine receptor antagonists are yet to show meaningful activity, CD39 is a marker of melanoma specific T_RM_ that will be explored later in this review (53, 54).

### Transcription factors

T_RM_ exhibit a distinct transcriptional program classically defined by the expression of Blimp-1 (*PRDM1*), Hobit (*ZNF683*) (55, 56) and RUNX transcription factor family 3 (*RUNX3*) (**Figure 2**) (57). This core program enables the maintenance of CD69 expression, while interfering with the expression of egress receptors such as S1PR1 and CCR7 (39). In addition, T_RM_ also express low, but residual levels of T-bet (*TBX21*), and suppression of Eomesodermin (*EOMES*) (terminal differentiation), Tcf1 (*TCF7*) and Krupel-like factor-2 (*KLF2*) (naïve/central memory phenotype) (58). T-bet and Eomes are T-box transcription factors which have key roles in driving T cell differentiation (59). The suppression of T-bet and Eomes is critical for T_RM_ development as it drives TGF-β signaling (38). In T_RM_, T-bet is maintained at residual levels to maintain IL-15 signaling to promote long-term survival (38). Eomes expression increases over time as CD8^+^ T cells undergo differentiation from naïve CD8^+^ T cells to T_EM_ and maintain homeostasis *via* IL-15 signaling (60). In T_RM_ however, Eomes expression is completely suppressed, as it is the KLRG1^-^CD127^+^ precursors that give rise to T_RM_ (61). This was demonstrated experimentally in a murine model of HSV where Mackay et al. demonstrated that after 4 weeks, skin T_RM_ completely suppressed *Eomes* expression, while maintaining a residual level of *TBX21* (38).

The transcriptional profile promoting T_RM_ differentiation and function is of translational interest. As mentioned previously, TGF-β is critical in the expression of CD103, but also plays a key role in the induction of Notch signaling in T_RM_ (62). Indeed, Notch (58), as well as nuclear receptor 4A1 (*NR4A1*) (63), are critical for the expression of CD69 and CD103 (64). The up-regulation of *RUNX3* also drives CD103 expression as its expression induces a profile of TGF-β response genes through remodeling of chromatin (57). Notch also induces transcription of IFN-γ mRNA, thereby influencing the cytotoxic capacity of T_RM_ (58). Transcriptional analysis of T_RM_ localized within the skin has also identified aryl hydrocarbon receptor (AhR) as a key transcriptional regulator promoting skin residency (65). In an AhR knockout model, no difference in CD8^+^ T cell infiltration was observed, but their survival decreased dramatically, indicating the role of AhR in long-term persistence (65).

### Immune checkpoint expression

T_RM_ often display a phenotype consistent with that of exhausted T cells with the expression of a range of immune checkpoints such as PD-1, LAG-3, T cell immunoreceptor with Ig and ITIM domains (TIGIT), OX-40, T-cell immunoglobulin and mucin-domain containing-3 (TIM-3) and LAG-3 (10, 27, 66–68) (**Figure 2**). Exhausted T cells arise from chronic antigen exposure during viral infections and cancer progression (69). When T cells transition to an exhausted phenotype, they lose their cytotoxic and proliferative functions with a decrease in production of IL-2, IFN-γ and TNF alongside an inability to degranulate against target cells (69). Gene expression data shows higher expression of immune checkpoints including *PDCD1*, *CTLA4*, *LAG3* and *TIGIT* by T_RM_ compared to T_CM_ or T_EM_ (66). PD-1 was among the first key markers that identified exhausted T cells in patients with chronic infections such as human immunodeficiency virus (70) and viral hepatitis (71). These seminal observations in viral immunology now form the basis of a large understanding of cancer immunity (72).

**Figure 3:**
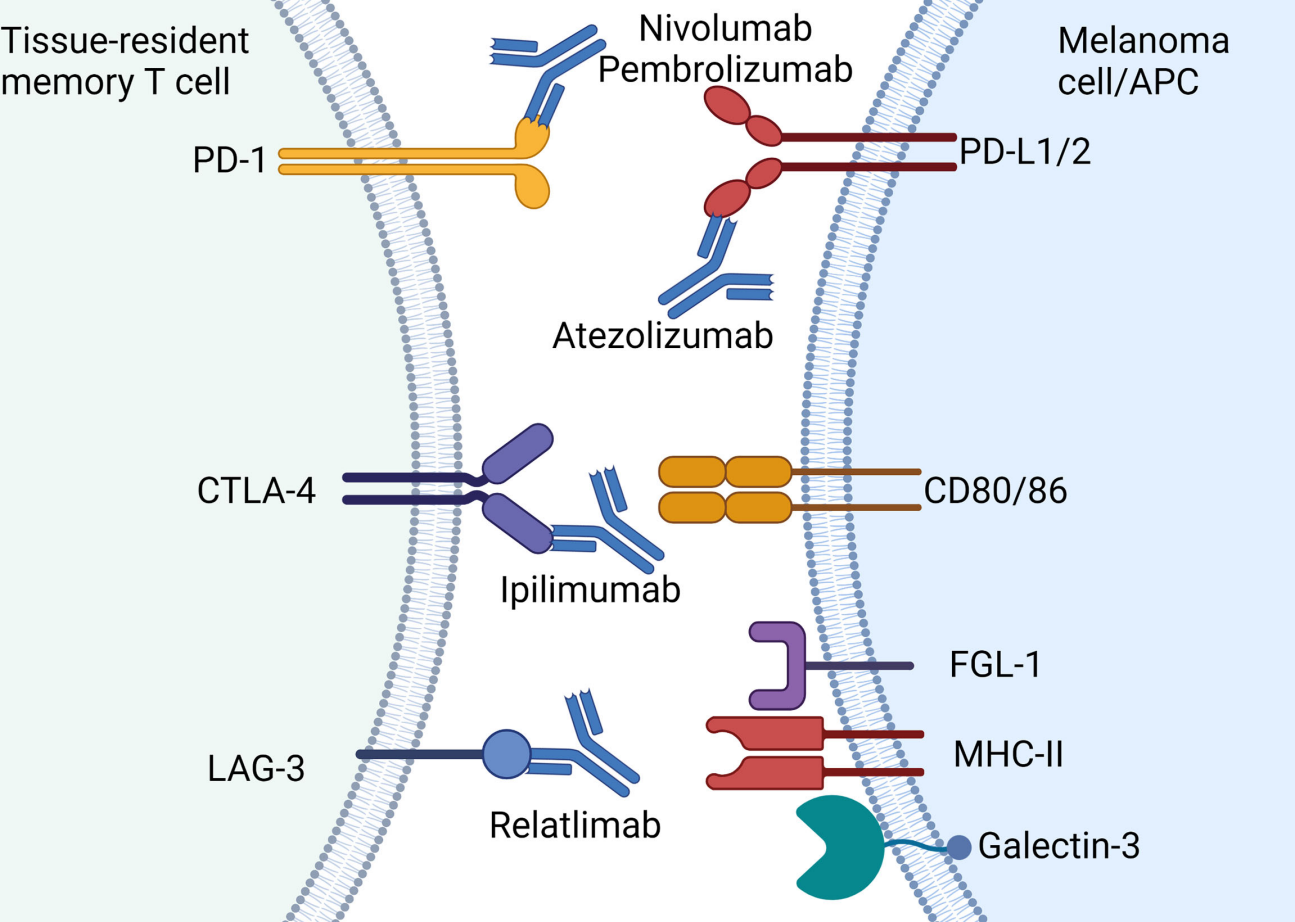
Current approved immune checkpoint inhibitors for the treatment of melanoma: Immune checkpoint molecules expressed by T_RM_ and the approved antibodies including PD-1 (nivolumab, pembrolizumab), PD-L1 (atezolizumab), CTLA-4 (ipilimumab) and LAG-3 (relatlimab). Note atezolizumab is only approved for use in combination with vemurafenib-cobimetinib. Created with Biorender.com.

Cancer cells that express PD-L1 that contact with CD8 bearing PD-1 leads to a signaling cascade that results in reduction of TCR signaling, proliferation and cytotoxicity that prevents anti-tumor activity (**Figure 3**) (73). Upon binding of PD-L1 to PD-1, phosphorylation of immune receptor tyrosine–based switch motif (ITSM) occurs, which binds to Src homology region 2 (SH2)-containing protein tyrosine phosphatase 2 (SHP2) (73). This directly inhibits ZAP70 thereby inhibiting TCR signaling whilst simultaneously downregulating both RAS and PI3K pathways that are required for downstream activation of critical transcription factors such as nuclear factor kappa-light-chain-enhancer of activated B cells (NF-κB) and nuclear factor of activated T cells (NFAT). Through these complex mechanisms, tumor cells that bear surface PD-L1 effectively halt cytotoxic immune responses by CD8 cells and therefore facilitates “immune escape.” Multiple studies show T_RM_ expression of PD-1 (10, 11, 74, 75) which indicates the cytotoxic potential of this cell type can be unleashed with anti-PD-1 antibodies such as nivolumab or pembrolizumab.

T_RM_ also express CTLA-4 which was the first immune checkpoint identified that was shown to inhibit anti-cancer responses by CD8 cells (76). Primarily CTLA-4 interactions occur at the time of antigen presentation between dendritic cells and T cells within the draining lymph node. T cell activation is dependent on co-stimulatory molecules namely CD80 and CD86 that bind to CD28. However CTLA-4 competes with CD28 thereby inhibiting costimulatory signaling which subsequently halts T cell activation and proliferation (77, 78). Under chronic TCR stimulation by either infection or malignancy, CTLA-4 expression becomes constitutive, dampening the immune response by induction of T cell anergy (79). T_RM_ from breast cancer samples displayed increased expression of *CTLA4* compared to T_EM_ populations (22). However, the role this key checkpoint plays with respect to T_RM_ function remains to be defined.

LAG-3 is highly expressed on skin T_RM_ and also acts as a negative regulator of T cell function (10). This type I transmembrane protein shares approximately 20% amino acid homology with CD4 but its specific mechanism of action and ligands remains unclear (80). LAG-3 migrates to the immunological synapse at the TCR-MHC-II junction and may competitively inhibit TCRs on CD8^+^ T cells with cross-presenting antigen presenting cells (81) (**Figure 3**). In addition to MHC class II, LAG-3 also binds with the lectin receptor galectin-3 and fibrinogen-like protein 1 (FGL1) (**Figure 3**) (82, 83). Upon binding of LAG-3 with galectin-3, IFN-γ production by CD8^+^ T cells is inhibited (82). While the exact mechanisms of LAG-3 remain controversial, this immune checkpoint has gained prominence with the development of relatlimab (anti-LAG-3 antibody) which has recently been shown to improve anti-tumor activity in combination with nivolumab (19).

Given T_RM_ widely express CTLA-4, PD-1 and LAG-3, which are inhibited by immune checkpoint inhibitors such as ipilimumab, nivolumab, pembrolizumab and relatlimab provides a strong biological rationale this CD8 subset is critical in facilitating anti-tumor responses in melanoma.

## T_RM_ are central in melanoma surveillance

Melanoma has been long regarded as an immunological cancer and the presence of tumor infiltrating lymphocytes is recognized as an important prognostic factor for survival (84). Clarke and colleagues described the first TIL classification system with absent, non-brisk and brisk categories (85). The brisk pattern is defined by the presence of lymphocytes throughout the entire tumor whereas non-brisk is typified by scattered, focal groups of lymphocytes which has been shown to be a prognostic factor of improved overall survival compared to absent TILs (86). Further refinement of a classification based upon TIL density and distribution by Azimi and colleagues showed survival in Stage III melanoma patients could be predicted (87). Efforts to refine TIL subsets using CD3, CD8 IHC has also been intensively investigated with greater CD3 and CD8 TIL being a strong predictor of survival (88).

Recent preclinical and translational studies have shown T_RM_ are the key TIL subset that facilitates immune control of melanoma. Using the B16 murine melanoma model, T_RM_ were shown to be essential to maintain melanoma in a state known as ‘immune-mediated equilibrium’ or melanoma dormancy (12). This phenomenon describes the process whereby melanoma cells are maintained as occult lesions *in situ* rather than continued growth and progression (89). Engraftment of pre-activated T cells were used to over-represent T_RM_ at the site, resulting in more efficient control from melanoma challenge. Using two-photon microscopy, T_RM_ were observed surveilling the skin in regions where melanoma cells were present. Depletion of T_CIRC_ demonstrated that T_RM_ were responsible for the protection from melanoma challenge, in a process driven by TNF-α. This study which substantiated T_RM_ are critical for maintaining immune-mediated equilibrium, may in part account for clinical observations of re-emerging melanoma after years of dormancy. Several human case studies which support the role of T_RM_ in immune control of melanoma have been observed. One case study showed occurrence of a secondary melanoma in breast tissue following a kidney transplant without a primary tumor on the recipient (90) as well as the recurrence of melanoma following 30 to 40 year disease-free periods (91–93), indicating long periods of immune control followed by escape. These data suggest that T_RM_ may play a role in human disease by maintaining occult lesions in the skin, sometimes for decades, before progressing to clinical disease.

However the precise mechanisms of immune escape with progression from “occult to overt” disease are currently unclear. Potentially other cells and factors such as T regulatory cells, myeloid derived suppressor cells and tumor associated macrophages (TAMS) could inhibit T_RM_ function and facilitate immune escape. The presence of TAM identified using CD68 or CD163 have an inverse relationship with survival in primary melanoma (94, 95) and may counter balance T_RM_ function. Moreover, as T_RM_ express PD-1, loss of antigen presentation machinery by melanoma is a well described mechanism of resistance to ICI and therefore may also contribute to immune escape (96, 97). In one study, downregulation of MHC class I, rather than complete loss was associated with de-differentiation of melanoma with increased expression of the receptor tyrosine kinase AXL and downregulation of micropthalmia-associated transcription factor (MITF) (98). Given the breadth of resistance mechanisms of ICI to melanoma including the innate PD-1 Resistance Signature (IPRES) (99, 100), T cell exclusion programs such as beta catenin (101), low tumor mutational burden (102), alternative immune checkpoint expression (103) amongst a host of others indicates the means of immune escape are also likely to be heterogenous.

Reinforcing the importance of T_RM_ facilitating immune control of melanoma is the central role these cells play in mediating vitiligo, an auto-immune condition leading to depigmentation of the skin. Patients with vitiligo bear melanocyte-specific CD8^+^ T cells that are involved with the destruction of melanocytes (104). Two separate groups using different B16 mouse models of vitiligo showed CD8^+^ T_RM_ identified by CD69^+^ CD103^+^ co-expression were critical in mediating vitiligo (105, 106). Moreover, both groups showed T_RM_ produced high levels of IFN-γ implicating their role in melanocyte destruction. Further human studies show CD49a^+^ CD103^+^ CD8^+^ (46) and CD69^+^ CD103^±^ CD8^+^ T_RM_ (105) were also central in mediating depigmentation with the latter study showing CXCR3 expression on melanocyte-specific T_RM_. The relationship of T_RM_ and vitiligo is of clinical importance as skin depigmentation is associated with improved clinical outcomes to anti-PD-1 treatment in metastatic melanoma (107, 108).

In addition, the immunological mechanisms that underpin vitiligo with responses to ICI has been recently demonstrated in an elegant study using patient matched melanoma tumor samples, blood and vitiligo affected skin that were subjected to TCR and single cell sequencing (109). Paired TCR sequencing of T_RM_ from the skin and T_EM_ from blood showed similar T cell clones that highly expressed IFN-γ which were maintained years after treatment with immunotherapy thereby indicating durable immunological memory of these subsets. Furthermore, three distinct populations of T_RM_ were identified using single cell sequencing: T_RM_-FOS, T_RM_-TOX and T_RM_-INFG each displaying different functional profiles. T_RM_-FOS consisted of a profile relating to TCR signaling but was not shown to correlate with improved patient outcomes. T_RM_-TOX displayed a more prototypical profile with high expression of immune checkpoints (LAG-3, PD-1 and CTLA-4), cytotoxicity (perforin and granzyme B) and key T_RM_ markers including CD103. The third cluster, T_RM_-IFNG expressed a strong cytokine profile with increased expression of IFN-γ, and in particular, TNF-α. Unlike T_RM_-FOS the T_RM_-TOX and T_RM_-IFNG populations were associated with improved survival. This data demonstrates that T_RM_ may be reactivated following ICIs and cause the onset of vitiligo, leading to improved patient outcomes. Importantly it highlights the importance of T_RM_ in maintaining long term host immunity which might partly account for the durable efficacy of immune checkpoint inhibitors in metastatic melanoma (4, 110).

The presence of T_RM_ transcriptional signatures are also associated with improved outcomes in various solid cancers such as breast cancer (22), endometrial cancer (24), ovarian cancer (23, 111) and melanoma (18, 112). Analysis of the Cancer Genome Atlas melanoma dataset using a T_RM_ core signature comprising of *CD69*, *ITGAE*, *CD8A*, *TNFSRF18*, *2B4* genes showed expression of T_RM_ related genes was associated with survival in an ICI naïve population (10). Furthermore, sophisticated computational analysis of the transcriptome of tumor infiltrating lymphocytes (TILs) from metastatic melanoma revealed better prognosis in patients with a T_RM_ signature compared to an early activation signature in those with poorer outcomes (66). Samples exhibiting this T_RM_ signature also showed greater dendritic cell activation and IFN-γ signaling, suggesting increased likelihood of response to anti-PD-1 therapy (66). Collectively, these transcriptional T_RM_ signatures from several groups provide further evidence to the integral role of T_RM_ in immune control of melanoma. Coupled with the high expression of immune checkpoints such as PD-1, CTLA-4 and LAG-3 further implicate T_RM_ as a key potential effector of response to immune checkpoint inhibitors. The next section details the clinical role of immune checkpoint inhibitors in the treatment of metastatic melanoma and translational studies that support T_RM_ as a critical mediator of response to ICI.

## Immune-checkpoint inhibitors in treatment of metastatic melanoma

Melanoma was the first tumor type where anti-tumor activity of ICIs, namely anti-CTLA-4 and anti-PD-1 antibodies, was demonstrated. Ipilimumab (anti-CTLA-4) was the first ICI developed and although objective responses were modest at 10-15% (113, 114), approximately 20% of patients demonstrated long term survival to 10 years (115). However, the advent of two anti-PD-1 antibodies, namely pembrolizumab and nivolumab, which were developed in parallel, revolutionized melanoma cancer therapeutics. The Phase III KEYNOTE-006 trial (116) compared two pembrolizumab regimens (10 mg/kg fortnightly; n = 279 or 3 weekly; n = 277) to ipilimumab (3mg/kg every 3 weeks for 4 doses; n = 278) which showed superior progression-free survival (PFS) and objective responses. The two combined pembrolizumab arms exhibited a response rate of 37% versus 13% in the ipilimumab control arm with a median PFS of 5.6 versus 2.8 months respectively (116). Accordingly median OS was 32.7 months versus 15.9 months in favor of pembrolizumab with similarly improved 5-year landmark survival rates at 38.7% compared to 31% for ipilimumab (110) (**Table 1**).

**Table 1:** Key clinical trials in metastatic/unresectable melanoma.

Trial/Year	Design (n)	Intervention	Stage (AJCC 7^th^ edition)	PFS	Response rate	Overall survival	Treatment related adverse events
MDX010-20 2010 (113)	Phase III (n=540)	Ipilimumab (3 mg/kg) + gp100 vaccine every 3 weeks for 4 doses (n=403) vs ipilimumab (3 mg/kg) every 3 weeks for 4 doses (n=137) vs gp100 vaccine (n=136)	Stage IV	Median PFS Ipilimumab-gp100 2.76 months (95% CI, 2.73 to 2.79) vs ipilimumab 2.86 months (95% CI, 2.76 to 3.02) vs gp100 2.76 months (95% CI, 2.73 to 2.83)	Ipilimumab-gp100 5.7% (3.7-8.4; p=0.04) vs ipilimumab 10.9% (6.3-17.4; p = 0.001) vs gp100 1.5%	Median OS 10.0 months ipilimumab-gp100 vs 10.1 months (95% CI, 8.0-13.8) ipilimumab vs 6.4 months (95% CI, 5.5-8.7) gp100 (0.2-5.2)	Grade 3-4 Ipi + gp100: 17.4% Ipilimumab: 22.9% gp100: 11.4%
CA184-024 2011 (114)	Phase III (n=502)	Ipilimumab (10 mg/kg) + dacarbazine (850 mg/m^2^) for 4 doses then dacarbazine monotherapy for another 4 doses (n=250) vs dacarbazine (850 mg/m^2^) (n=252)	Stage IV	Not reported	Ipilimumab + dacarbazine 15.2% vs dacarbazine 10.3%	Median OS Ipilimumab-dacarbazine 11.2 months (95% CI 9.4 to 13.6) vs dacarbazine 9.1 months (95% CI, 7.8-10.5)	Grade 3-4 any cause AE Ipilimumab-Dacarbazine: 56.3% Dacarbazine: 29.4%
CHECKMATE-067 2015 (1, 117)	Phase III (n=1296)	Ipilimumab (3 mg/kg) + nivolumab (1 mg/kg) every 3 weeks for 4 doses then nivolumab (n=314) vs nivolumab (3mg/kg) every 2 weeks (n=316) vs ipilimumab (3mg/kg) every 3 weeks for 4 doses + placebo (n=315)	Unresectable Stage III or IV	Median PFS Ipi-nivo 11.5 months (95% CI, 8.7 to 19.3) vs nivolumab 6.9 months (95% CI, 5.1 to 9.7) vs ipilimumab 2.9 months (95% CI, 2.8 to 3.2) Ipi-nivo vs ipi HR=0.42, (95% CI, 0.35 to 0.51) p< 0.0001 Nivolumab vs ipi HR=0.53, (95% CI 0.44 to 0.64, p< 0.0001 Ipi-nivo vs nivo HR= 0.79, (95% CI, 0.65 to 0.97) (*post hoc*)	Ipi-nivo 58% vs nivolumab 44% vs ipiliumumab 19%	Minimum follow-up of 6.5 years: median OS ipi-nivo 72.1 months (38.2-NR) vs nivolumab 36.9 months (28.2-NR) vs ipilimumab 19.9 months (16.8-24.6)	Grade 3-5 Ipi-nivo: 59% Nivolumab: 21% Ipilimumab: 28%
KEYNOTE-006 2015 (116)	Phase III (n=834)	Pembrolizumab (10 mg/kg) every 2 weeks (n=279) or every 3 weeks (n=277) vs ipilimumab 3 mg/kg every 3 weeks for 4 doses (n=278)	Unresectable stage III or IV	Median PFS Combined pembrolizumab groups 8.4 months (95% CI, 6.6–11.3) vs ipilimumab 3.4 months (2.9–4.2) HR=0.70, (95% CI, 0.48–0.67), p<0.0001	Pembrolizumab 33.7% every 2 weeks (p<0.001 vs ipilimumab), pembrolizumab every 3 weeks 32.9% (p<0.001) vs 11.9% for ipilimumab	Median OS Combined pembrolizumab groups 32.7 months (95% CI, 24.5–41.6) vs ipilimumab 15.9 months (95% CI, 1.3–22.0) HR=0.73, (95% CI, 0.61–0.88), p=0.00049	Grade 3-5 Pembrolizumab every 2 weeks: 13.3% Pembrolizumab every 3 weeks: 10.1% Ipilimumab: 19.9%
CA184-169 2017 (118)	Phase III (n=727)	Ipilimumab (10 mg/kg) (n=365) vs ipilimumab (3 mg/kg) (n=362) every 3 weeks for 4 doses	Unresectable stage III or IV	Median PFS Ipilimumab 10 mg/kg 2.8 months (95% CI, 2.8–3.0) vs ipilimumab 3 mg/kg 2.8 months (2.8–2.8) HR=0.89 (95% CI, 0.76–1.40), p=0·16	Ipilimumab 10 mg/kg 15% (11.8-19.5) 10mg/kg vs ipilimumab 3mg/kg 12% (9.0=-16.0)	Median OS Ipilimumab 10 mg/kg 15.7 months (95% CI, 11.6 to 17.8) vs Ipilimumab 3 mg/kg 11.5 months (95% CI, 9.9 to 13.3) HR=0.84, (95% CI, 0.71 to 0.99), p=0.04	Grade 3-5 Ipilimumab (10mg/kg): 43% Ipilimumab (3 mg/kg): 18%
RELATIVITY-047 2022 (19, 119)	Phase II/III (n=714)	Relatlimab (160 mg) + nivolumab (480 mg) every 4 weeks (n=355) vs nivolumab (480 mg) every 4 weeks (n=359)	Unresectable stage III or IV*	Median PFS Rela-nivo 10.1 months (95% CI, 6.4 to 15.7) vs nivolumab 4.6 months (95% CI, 3.4 to 5.6) HR=0.78, (95% CI, 0.64-0.94)	Rela-nivo 43.1% (95% CI, 37.9-48.4) vs nivolumab 32.6% (95% CI, 27.8-37.7)	Median OS not reached for rela-nivo vs nivolumab 34.1 months HR=0.80, (95% CI, 0.64-1.1) p = 0.0593	Grade 3-5 Rela-nivo: 19.7% Nivolumab: 10.3%

ipi-nivo, combination ipilimumab-nivolumab; ORR, overall response rate; HR, hazard ratio; TRAE, treatment related adverse events; rela-nivo, combination relatlimab-nivolumab; *Enrollment based upon AJCC 8^th^ edition.

Table lists trials and the year they were conducted, trial design and intervention, stage of disease and key outcomes such as progression-free survival (PFS), response rates, overall survival (OS) and any significant toxicity. Stage as per American Joint Committee on Cancer (AJCC) 7^th^ edition unless otherwise noted.

Further evidence for first line anti-PD-1 monotherapy was demonstrated in the CHECKMATE-067 randomized control trial which investigated nivolumab (n=316) or combination ipilimumab-nivolumab (n=314) versus ipilimumab monotherapy (n=315) (1). Response rates of 57.6%, 43.7% and 19% for ipilimumab-nivolumab, nivolumab monotherapy and ipilimumab monotherapy respectively were observed. Accordingly median PFS was 11.5 months for combination ipilimumab-nivolumab, 6.9 months for nivolumab and 2.9 months for ipilimumab. The hazard ratio (HR) for PFS for combination ipilimumab-nivolumab was 0.42 versus ipilimumab and 0.53 for nivolumab compared to ipilimumab indicating a substantial improvement over anti-CTLA-4 monotherapy.

Whilst the trial was not designed nor powered to formally compare combination ipilimumab-nivolumab over nivolumab monotherapy, the *ad hoc* HR for PFS of these two arms was 0.79 (95% CI 0.64-0.96) in favor of the combination (4). This translated to a moderate 7% absolute improvement in PFS at the five-year landmark in favor of the combination versus nivolumab monotherapy (36% vs 29%). Whilst ipilimumab-nivolumab may appear to have superior efficacy over anti-PD-1 monotherapy, the combination was less tolerable with treatment related grade 3-4 (severe to life threatening) adverse events of 59% compared to 23% with nivolumab.

Combination anti-LAG3 with nivolumab however has emerged as a more tolerable ICI doublet regimen (19). In the Phase II/III Relativity-047 trial combination relatlimab with nivolumab demonstrated superior median PFS of 10.1 months compared to 4.6 months for nivolumab monotherapy (HR=0.75; 95%CI 0.62-0.92) (**Table 1**). Landmark PFS at 12 months was 47.7% for relatlimab-nivolumab compared to 36% for nivolumab monotherapy. Objective response rates were 43.1% for relatlimab-nivolumab and 32.6% for nivolumab monotherapy (119). To date superior overall survival for the anti-LAG3 combination is yet to be shown with median overall survival not yet attained for relatlimab-nivolumab and 34.1 months for nivolumab monotherapy (HR=0.80; 95%CI 0.64-1.01, p=0.0593). Importantly the toxicity profile was encouraging with grade 3-4 treatment-related adverse events (TRAE) of 18.9% in the combination arm which compares favorably to ipilimumab-nivolumab. Hence combination anti-LAG3 and anti-PD1 may become the immune checkpoint inhibitor regimen of choice in metastatic melanoma.

### T_RM_ as effectors of immune checkpoint inhibitors

Both KEYNOTE-006 and CHECKMATE-067 established anti-PD-1 as the mainstay treatment for metastatic melanoma which also led to studies investigating the tumor microenvironment and circulating immune subsets associated with resistance or response to therapy. Previous studies showed anti-PD-1 anti-tumor responses were reliant on the localization of CD8^+^ T cells at the tumor margin (8). Efforts were then aimed at characterization of the CD8^+^ T cell subset associated with response to ICI. One study showed intratumoral T_EM_, identified by CD8^+^CD4^-^CD45RO^+^, were expanded in patients treated with pembrolizumab (n=53) (120). In keeping with a “T_RM_ like” phenotype, CCR7 was not expressed by this subset. Other cell populations such as T_REG_, natural killer and monocytes did not appear to be expanded during PD-1 treatment. Although, this study did not utilize key T_RM_ markers such as CD103 or CD69 in their analyses it did establish memory T cells as being crucial in mediating responses to anti-PD-1.

Further studies by Boddupalli and colleagues demonstrated T_RM_ as a key CD8^+^ T cell population involved with ICI response (11). Using single cell mass cytometry, comprehensive analyses of TIL from metastatic lesions showed approximately 60% of CD8^+^ T cells exhibited a T_RM_ phenotype with CD45RO^+^CD69^+^CCR7^–^ expression. Further analyses showed approximately 50% of these cells expressed CD103, further demonstrating the T_RM_ phenotype. The variable expression of this key T_RM_ marker is likely due to the heterogeneity of anatomical sites which included skin, soft tissue, lymph node, colon and lung that were analyzed. These T_RM_ also exhibited high expression of immune checkpoints, namely PD-1 and TIM-3. Gene expression data of sorted CD8^+^CD69^+^ T cells also displayed enrichment of NR4A2, CTLA-4, SKI-like proto-oncogene (SKIL), regulator of G protein signaling 1 (RGS1) with low expression of CCR7 and S1PR1, thereby reinforcing the identification of the T_RM_ as a prevalent T cell subset in metastatic tissue in this study.

Separately Edwards et al, provided additional evidence for T_RM_ as being a mediator of ICI responses (10). Utilizing multiplex IHC of pre-treatment and early on treatment biopsies of melanoma patients treated with ICI showed that CD8^+^CD103^+^ cells were increased in patients that exhibited an objective response to treatment (10). Supporting the findings from Boddupalli et al. (11), analysis of selected melanoma deposits by flow cytometry demonstrated approximately 30% of CD8^+^ TILs co-expressed CD69 and CD103, consistent with a T_RM_ phenotype in ICI naïve patients. Moreover expression of immune checkpoints such as PD-1 and LAG-3 were expressed highly in CD8^+^CD69^+^CD103^+^ T cells compared to CD8^+^CD69^-^CD103^-^ cells (10).

In the largest cohort to date aimed at identifying immune cell populations involved with response to anti-PD-1 (n=63) or combination anti-CTLA-4 and anti-PD-1 in melanoma (n=57) Gide et al. identified a T effector memory signature was associated with response to ICI (103). Transcriptome data showed IFN-γ related genes and T cell infiltrating genes such as *CD8A*, *CD8B*, *ITGAE* (CD103), *PDCD1* (PD-1) were associated with response and improved PFS with anti-PD-1 therapy. Utilizing mass cytometry from patient samples collected at baseline and early on treatment, an EOMES^+^CD69^+^CD45RO^+^ effector memory T cell phenotype was identified that correlated with response to ICI (103). Again similar to the findings of Boddupalli (11), approximately 50% of these cells expressed CD103 with very low CCR7 expression indicating a T_RM_ phenotype is involved. The high EOMES expression is not a typical feature of T_RM_. However, as the tumor biopsies were acquired at an early timepoint approximately 2-weeks after initiation of ICI treatment these cells potentially represents an immature T_RM_ differentiation program. In summary, these translational datasets using a combination of gene expression, immunofluorescence and mass cytometry demonstrates T_RM_ as a key CD8 subset in the tumor microenvironment that mediates anti-tumor response to ICI.

## Adjuvant treatment for stage II and III melanoma

Given the impressive long-term survival in advanced disease, adjuvant anti-PD-1 was quickly investigated in patients following surgical excision of the primary melanoma and regional lymph node basin in stage III melanoma. The first trial using adjuvant anti-PD-1 in melanoma was CHECKMATE-238 (121) which compared adjuvant nivolumab 3 mg/kg (n=453) for 12 months to ipilimumab 10 mg/kg (n=453) in resected stage IIIB, IIIC or IV disease. Ipilimumab (10 mg/kg 3 weekly for 4 doses then every 12 weeks) was the comparator arm as it had previously been shown to improve recurrence rates and OS in the EORTC 18071 trial (122). However, given the high grade 3-4 toxicity rate of approximately 55%, adjuvant ipilimumab was not used widely. As expected, nivolumab showed superior recurrence-free survival (RFS) (HR=0.65; 97.56% CI 0.51-0.83) compared to ipilimumab (121). Updated follow-up of Stage IIIB and IIIC melanoma confirmed the superiority of nivolumab compared to ipilimumab (HR=0.71; 95% CI 0.58-0.88) with a 4-year landmark RFS of 51.7% versus 41.2% in favor of PD-1 monotherapy. Importantly nivolumab was more tolerable with treatment related grade 3-4 adverse events of 14.4% compared to 45.9% for ipilimumab. As such anti-PD-1 heralded a new benchmark in adjuvant therapy for melanoma (123) (**Table 2**).

**Table 2:** Clinical trials investigating adjuvant ICIs in resected melanoma.

Trial/Year	Design (n)	Stage/Group(AJCC 7^th^ edition)	Intervention (n)	RFS	DMFS	Overall survival	Treatment related adverse events
EORTC-18071 2015 (122)	Phase III (n=951)	Resected High-risk stage III	Ipilimumab (10mg/kg) every 3 weeks for 3 years (n=475) vs placebo (n=476)	Median RFS Ipilimumab 26.1 months (95% CI 19.3-39.3) vs placebo 17.1 months (95% CI 13.4-21.6) HR=0.75, (95% CI, 0.64-0.90), p=0.0013	5-year DMFS Ipilimumab 48.3% vs 38.9% placebo HR=0.75, (95.8% CI, 0.64-0.92), p=0.02	5-year OS Ipilimumab 65.4% vs placebo 54.4% HR=0.72, (95.1% CI, 0.58-0.88), p =0.001)	Grade 3-5 Ipilimumab: 55% Placebo: 26%
CHECKMATE-238 2017 (121)	Phase III (n=906)	Resected stage IIIB, C or IV	Nivolumab 3mg/kg every 2 weeks for 1 year (n=453) vs ipilimumab 10mg/kg every 3 weeks for four doses then 12 weeks for 1 year (n=453)	4-year RFS Nivolumab 51.7% (95% CI 46.8–56.3) vs ipilimumab 41.2% (36.4–45.9) HR=0.71, (95% CI, 0.60–0.86), p=0.0003	Stage III 4-year DMFS Nivolumab 59% vs ipilimumab 53% HR=0.79, (95% CI, 0.63-0.99)	4-year OS Nivolumab 77.9% (95% CI, 73.7–81.5) vs ipilimumab 76.6% (72.2–80.3) HR=0.87, (95% CI, 0.66–1.14]	Grade 3-5 Nivolumab: 14.4% Ipilimumab: 45.9%
COMBI-AD 2017 (124, 125)	Phase III (n= 970)	Resected stage III	Dabrafenib (150 mg bd) + trametinib 2 mg od for 12 months (n=438) vs placebo (n=432)	5-year RFS Dabrafenib-trametinib 52% (95% CI, 48-58) vs placebo 36% (95% CI, 34-43) HR=0.51, (95% CI, 0.42-0.61)	5-year DMFS Dabrafenib-trametinib 65% vs placebo 54% HR=0.55, (95% CI, 0.44-0.70)	Not reported	Grade 3-4 any cause AE Dabrafenib-trametinib: 41% Placebo: 14%
KEYNOTE-054 2018 (5, 126)	Phase III (n=1019)	Resected stage III	Pembrolizumab 200 mg every 3 weeks (n=513) vs placebo for 18 cycles (1 year) (n=505)	5-year RFS Pembrolizumab 55.4% (95% CI, 50.8-59.8) vs placebo 38.3% (95% CI, 33.9-42.7) HR=0.61, (95% CI, 0.51-0.72), p=0.04	5-year DMFS Pembrolizumab 60.6% (95% CI 56.0-64.9) vs placebo 44.5% (95%CI, 39.9-48.9) HR=0.62, (95% CI, 0.52-0.75), p=0.04	Not reported	Grade 3-5 Pembrolizumab: 31.6% Placebo: 18.5%
CHECKMATE-915 2020 (127)	Phase III (n=1844)	Resected stage IIIB - IIID or IV*	Adjuvant ipilimumab 1mg/kg every 6 weeks + nivolumab 240mg every 2 weeks (n=920) vs Nivolumab (480mg) every 4 weeks (n=924)	2-year RFS Ipi-nivo for Stage III 64.6% nivolumab 63.6%. HR=0.94, (95% CI, 0.80-1.11)	Stage III disease DMFS ipi-nivo 75.4% vs nivolumab 77.4% HR=1.01, (95% CI, 0.83-1.23)	Stage III disease 2-year OS ipi-nivo 77.4% vs nivolumab 75.4% HR=1.01, (95% CI, 0.83-1.23)	Grade 3-5 Ipi-nivo: 33.0% Nivolumab: 13.0%
KEYNOTE-716 2022 (128, 129)	Phase III (n=976)	Resected stage IIB or IIC*	Pembrolizumab 200 mg every 3 weeks (n=487) vs placebo for 18 cycles (1 year) (n=489)	2-year DMFS Pembrolizumab 81.2% vs placebo 72.8% HR=0.64, (95% CI, 0.50-0.84)	2-year DMFS pembrolizumab 88.1% vs placebo 82.2% HR=0.64, (95% CI, 0.47-0.88), p=0.0029	Not reported	Grade 3-5 Pembrolizumab: 26% Placebo: 17%

TRAE, treatment-related adverse event; ipi-nivo, combination ipilimumab-nivolumab *Enrollment based upon AJCC 8^th^ edition.

Table lists trial name and year, trial design and intervention, stage of disease and patient groups and trial outcomes such as recurrence free survival (RFS), distant metastases free survival (DMFS), overall survival (OS) and treatment related adverse events. Stage as per American Joint Committee on Cancer (AJCC) 7^th^ edition unless otherwise noted.

Shortly thereafter the KEYNOTE-054 trial investigated a 12-month course of pembrolizumab (200 mg, 3 weekly) (n=513) compared with placebo control (n=505) in resected stage III melanoma (5). Pembrolizumab reduced recurrences by approximately 40% (HR=0.57; 98.4% CI 0.43-0.74; p<0.001) compared to placebo (5). Distant metastasis free survival (DMFS), a secondary endpoint of this study reported after 5 years was 60.6% in the pembrolizumab arm compared to 44.5% for placebo which strongly justifies anti-PD-1 in this setting (HR=0.62; 95% CI 0.52-0.74) (126). Treatment related grade 3-4 adverse events for pembrolizumab were in line with nivolumab in CHECKMATE-238 at 14.7% with 13.8% of patients discontinuing anti-PD-1 due to toxicity (**Table 2**).

In addition to anti-PD-1, combination BRAF-MEK inhibitors namely dabrafenib-trametinib are also effective in reducing recurrences in melanoma. Approximately 40% of melanoma harbor a BRAF^V600^ mutation which is amenable to sequential blockade of the mitogen activated protein kinase pathway with BRAF-MEK inhibitors (130, 131). While the anti-tumor activity of BRAF-MEKi are attributable to inhibition of the BRAF^V600^ oncogene, this targeted therapy also aids in recruitment of CD8 into the TME (132, 133) *via* several immunomodulatory properties including enhancement of melanoma differentiation antigens (132, 134, 135) and MHC Class I expression (134–136) alongside reduction of immunosuppressive cytokines such as IL-6 with IL-10 (132, 137). Whether BRAF-MEKi also enhances T_RM_ establishment is currently unclear. In the metastatic setting, combination BRAF-MEK inhibitors are associated with high response rates of 60-70% with median PFS of approximately 11-12 months (138, 139). The COMBI-AD Phase III trial had a similar study design to KEYNOTE-054 which investigated 12 months of dabrafenib-trametinib (n=438) compared to placebo (n=432) in resected stage III melanoma (124). Dabrafenib-trametinib improved RFS with a hazard ratio of 0.57 (95% CI; 0.39–0.58) with an improved 5-year RFS rate of 52% compared to 36% in the placebo arm (125). DMFS at 5 years was also superior for dabrafenib-trametinib at 65% compared to 54% in placebo (HR for distant metastasis or death=0.55; 95% CI; 0.44 to 0.70). Interestingly 25% of patients discontinued dabrafenib-trametinib due to toxicity which is somewhat higher compared to adjuvant anti-PD-1 monotherapy (10-15%). Dabrafenib-trametinib induces drug induced pyrexia which necessitates dose interruption and in some instances discontinuation.

Although anti-PD-1 reduces recurrences in Stage III melanoma, approximately 45% of patients still recur despite adjuvant treatment (126). Given the improved response rate of combination ipilimumab-nivolumab in the metastatic setting, the CHECKMATE-915 (127) trial investigated this immunotherapy doublet compared to nivolumab monotherapy to reduce recurrences in Stage III melanoma (**Table 2**). Ipilimumab was administered at a lower dose of 1 mg/kg (six weekly) and nivolumab 240 mg (two weekly) for 1 year in the combination arm as tolerability is dose dependent (118). Unfortunately, combination therapy resulted in higher treatment related grade 3-4 adverse events at 33% compared to nivolumab at 13% with no difference in the primary endpoints of RFS (HR=0.92; 97.3% CI, 0.77-1.09); P = 0.26) in the adjuvant setting. As such BRAF-MEK inhibitors or anti-PD-1 monotherapy remain the standard of care for adjuvant treatment in Stage III melanoma.

Building upon the improvements in stage III melanoma, anti-PD-1 has also been investigated to reduce recurrences in Stage II disease. Stage IIB (melanoma thickness < 2.0 – 4.0 mm with ulceration or melanoma thickness > 4.0 mm without ulceration) and IIC melanoma (melanoma thickness > 4.0 mm with ulceration) exhibit 5-year overall survival outcomes of 87% and 82% respectively (140). By comparison Stage IIIB melanoma exhibits 5-year survival of 83% which provides a strong clinical rationale for adjuvant trials particularly in IIC disease. The Phase III KEYNOTE-716 trial compared 1 year of pembrolizumab (200 mg IV 3 weekly) treatment with placebo in resected stage IIB and IIC melanoma (128). Notably 64% (625 out of 976) of enrolled patients had Stage IIB melanoma. In the second interim analysis with a median follow-up period of 20.9 months, recurrences in pembrolizumab treated patients was lower at 15% compared to 24% in the placebo control arm (HR=0.61; 95% CI 0.45–0.82). Treatment related grade 3-4 adverse events in the pembrolizumab arm were in line with prior adjuvant studies at 16%. A further update of the trial with a median follow-up of 37.2 months showed 2 year RFS at 81.2% in the pembrolizumab arm compared to 72.8% for placebo (129). Notably distant metastasis free survival was in favor of pembrolizumab at 88.1% compared to 82.2%. Given this moderate improvement in distant metastasis free survival balanced with the side effect of treatment, further follow-up of the trial is required to ascertain overall potential benefit. In addition, the results of CHECKMATE-76K (NCT04099251) are awaited which investigates nivolumab and may clarify the role of adjuvant anti-PD1 in Stage II disease.

### T_RM_ in primary and stage III melanoma

As mentioned previously, TILs are a well-known prognostic factor for melanoma, but only until recently have T_RM_ come to the forefront as a specific CD8 subset mediating immune control. Two recent studies utilizing multiplex immunohistochemistry investigated subpopulations of TILs and how they are linked to patient outcomes (53, 54). The first study quantified various populations of intra-tumoral CD8^+^ T cells in samples from patients with Stage III melanoma prior to adjuvant treatment with anti-PD-1 or combination anti-PD-1 with anti-CTLA-4 on clinical trial (54). Patients who benefited from adjuvant anti-PD-1 harbored greater proportions of CD39^+^ CD103^+^ PD-1^+^ CD8^+^ T cells with improved RFS. Conversely patients exhibiting a greater proportion of “bystander” cells delineated by CD39^-^CD103^-^PD-1^-^CD8^+^ had comparatively worse outcomes. Moreover the CD39^+^ CD103^+^PD-1^+^CD8^+^ were located closer to the tumor margin in comparison to their bystander counterparts which further implicates their role in clinical benefit of adjuvant ICI.

Following up their work in Stage III melanoma, a similar multiplex immunohistochemistry platform was utilized for studies in primary melanomas measuring at least 1 mm thick (53). A T_RM_ population delineated by CD39^+^CD103^+^PD-1^-^CD8^+^ was found in high proportions as both stromal and intratumoral CD8 T cells. Moreover, this population of cells were shown to have improved recurrence-free survival (RFS) ≥5 years and were also localized to the tumor margin. It is interesting to speculate whether this population of T_RM_ may represent a potential biomarker for clinical benefit for adjuvant anti-PD-1 in stage II melanoma. However, the technical challenges of multiplex immunohistochemistry reserve this as an investigational tool and not currently for clinical use.

Altogether these two published studies further implicate CD39^+^ as a key T_RM_ biomarker of tumor-reactive cells and as a potential biomarker of clinical benefit for ICI. Notably the key T_RM_ markers CD69 and CD49a were not included in these studies which would be useful to further define their phenotype. Further confirmatory studies are required to establish CD39 as a melanoma specific T_RM_ marker and to elicit the role of other cell types in the tumor microenvironment that shapes the immune response.

## Future directions: Adjuvant and neoadjuvant treatment for melanoma

Although adjuvant anti-PD-1 or BRAF-MEK inhibitors reduce the risk of recurrence in Stage II and III melanoma, much work is required to further improve outcomes. As mentioned previously approximately 45% of patients relapse despite adjuvant anti-PD-1 in Stage III melanoma (126). In KEYNOTE-054, the impact of recurrence free survival from pembrolizumab was mostly attributed to a reduction in distant metastases from 53.3% in the placebo group compared to 39.1% for the anti-PD-1 group (126). Adjuvant pembrolizumab reduced the rate of lung (13.2% pembrolizumab arm versus 21.0% placebo), lymph node (12.6% vs 18.0%) and liver (7.8% versus 11.3%) as sites of distant relapse compared to placebo (126). Interestingly pembrolizumab did not greatly reduce local or local regional relapses as the first site of recurrence at 14.4% in the pembrolizumab group and 19.0% in the control arm. This observation highlights the need to target the skin tumor microenvironment to reduce the likelihood of local recurrences. As outlined above, LAG-3 is expressed on skin T_RM_ and therefore blockade of this immune checkpoint might be beneficial in reducing such local recurrences. The RELATIVITY-098 trial (NCT05002569) investigates adjuvant relatlimab-nivolumab in resected stage III melanoma. The improvement in PFS and a favorable toxicity profile shown in the previously mentioned RELATIVITY-047 trial warrants investigation of relatlimab in the both the adjuvant and neoadjuvant (NCT02519322) setting.

The focus of the treatment paradigm in melanoma has shifted earlier to neoadjuvant therapy for stage III disease (6). Neoadjuvant systemic therapy allows histopathological assessment of response to treatment which may allow personalization of post-surgical adjuvant management. A pooled analysis of six neoadjuvant melanoma trials including anti-PD-1 monotherapy (n=37), combination ipilimumab-nivolumab (n=104) as well as BRAF-MEK inhibitors (n=51) in the neoadjuvant setting suggested pathological complete response (pCR) is a reasonable surrogate endpoint for relapse free survival. In this analysis after a median follow-up of 20.9 months, patients who achieved pCR with neoadjuvant treatment demonstrated superior RFS than in patients who did not have a pCR (89% versus 50% at 2 years; p<0.001). Interestingly patients who had a near pCR or partial pathological response (pPR) from immunotherapy had similar 2-year RFS that exceeded 90% as with pCR. Conversely patients treated with targeted therapy who did not achieve pCR (including pPR) had poor outcomes (2-year RFS for pCR of 79% vs non-pCR of 13%), similar to the cohort with pathological no response (pNR) after treatment. The 2-year RFS was 96% for patients treated with immunotherapy who achieved pCR, near pCR or even partial pathological response. Given the encouraging RFS observed with patients who exhibited a pCR or near pCR, neoadjuvant treatment for Stage III melanoma is an area of intensive investigation.

The pivotal OpACIN Phase II dose finding study (n=99) demonstrated combination ipilimumab-nivolumab with two cycles of ipilimumab 1 mg/kg plus nivolumab 3 mg/kg (Arm B) was better tolerated than two cycles of ipilimumab 3 mg/kg plus nivolumab 1 mg/kg once every 3 weeks (Arm A) or two cycles of ipilimumab 3 mg/kg once every 3 weeks directly followed by two cycles of nivolumab 3 mg/kg once every 2 weeks intravenously (Arm C) (7). Treatment related grade 3-4 adverse events was 20% for Arm B compared to 38% and 50% for arms A and C respectively. Importantly pathological complete response with the ipilimumab 1 mg/kg dose (Arm B) was very encouraging at 57%.

Based upon the OpACIN results, the PRADO phase II study (n=99 investigated 2 doses of combination ipilimumab 1 mg/kg and nivolumab 3 mg/kg in stage IIIB-IIID melanoma followed by response directed adjuvant therapy (8). Response assessments were performed after six weeks of neoadjuvant ICI and resection of the index lymph node (ILN) that was defined as the largest lymph node metastasis at baseline. Patients who achieved a major pathological response (≤10% viable tumor) in the ILN, did not proceed with therapeutic lymph node dissection (TLND) nor adjuvant therapy. Patients with a partial pathological response (pPR defined by >10% to ≤50% viable tumor) or pathological non-response (pNR ≥50% viable tumor) proceeded to TLND alone and TLND followed by adjuvant systemic therapy respectively. The primary objective of pathological response rate (pRR) was achieved in 72% (95% CI; 62-80%) of patients with a major pathological response (MPR) in 61% (95% CI; 50-70%). At two years both the RFS and distant metastasis free survival was 64% in patients with a pPR, compared with 93% and 98% in major pathological response respectively. Highly encouraging RFS and DMFS rates at 2 years were maintained at 85% and 89% respectively for all patients who underwent surgery. This approach may potentially lead to reduced surgical and treatment related morbidity without compromising on longer term clinical outcomes. However results of longer term follow up are awaited.

In addition to ipilimumab-nivolumab, combinations of targeted therapy and anti-PD-1 in the neoadjuvant setting have also been investigated. The NeoTrio phase II study investigated stage III BRAF V600 mutant melanoma treated with six weeks of neoadjuvant pembrolizumab monotherapy or in combination with dabrafenib-trametinib (D-T) in two different regimens (141). Patients were randomized 1:1:1 to pembrolizumab (n=20), sequential D-T followed by pembrolizumab (n=20) or concurrent D-T with pembrolizumab (n=20). Neoadjuvant therapy was followed by total lymph node dissection and adjuvant systemic therapy. Preliminary results showed pathological complete responses pCR were highest in concurrent therapy (50%) compared to pembrolizumab monotherapy (30%) and sequential therapy (15%). Rates of MPR for concurrent, pembrolizumab monotherapy and sequential therapy were 55%, 40% and 30% respectively. Landmark 12 month RFS and event free survival was similar across all three groups. However longer term follow up is required to ascertain the influence of pathological response on rates of disease recurrence.

In conclusion, the use of pre-operative immune checkpoint inhibitors for stage III melanoma are under intensive investigation and may improve upon the benchmarks set in the adjuvant resected setting. The side effect of ICI treatment is an important consideration, but the neoadjuvant platform shows very promising relapse free survival. Well characterized T_RM_ pre-clinical models of cutaneous melanoma may also inform neoadjuvant approaches as it provides opportunities to assess both the tumor and lymph node responses to ICI simultaneously as well as test new immunotherapy combinations. Moreover, the recent advent of spatial transcriptomics may provide further insights into the mechanisms of ICI on T_RM_ function alongside assessment of the TME in these pre-clinical neoadjuvant models. Future neoadjuvant clinical studies should include biomarker assessment for clinical benefit but also consideration of the immunological roles T_RM_ play given the recent translational studies highlighting the importance of this subset in both early and metastatic melanoma.

## Concluding remarks

The development of immune checkpoint inhibitors has fundamentally changed the systemic management of metastatic cutaneous malignancies and are now employed in earlier lines as adjuvant treatment in Stage II and III melanoma. T_RM_ are implicated in facilitating anti-melanoma immune responses, providing enhanced tumor control and promoting the phenomenon of “immune-mediated equilibrium”. Importantly T_RM_ express an array of immune checkpoints such as PD-1, CTLA-4 and LAG-3, which are all current targets of ICIs in melanoma. Therefore, we propose that T_RM_ are prominent mechanistic targets for ICIs, and the clinical success of these therapies depend on the activity of these cells at the tumor site. Future studies are required to fully unveil the role T_RM_ play in the anti-tumor response, and more importantly how this can be exploited in future therapeutic strategies particularly in the neoadjuvant setting.

## Author contributions

KP, A-JI and PL contributed to the conception of the manuscript. KP, A-JI and PL wrote the first draft of the manuscript. JA, AM, JW and PL provided critical appraisal of the manuscript. All authors contributed to the article and approved the submitted version.

## Conflict of interest

PL has received honoraria from Bristol Myers Squibb and Pfizer. PL received institutional funding for the purposes of clinical trials from Ambrx, AstraZeneca, Beigene, Bristol Myers Squibb, Pimera, MSD and Roche Genentech. These companies did not provide funding for the publication of this article.

The remaining authors declare that the research was conducted in the absence of any commercial or financial relationships that could be construed as a potential conflict of interest.

## Publisher’s note

All claims expressed in this article are solely those of the authors and do not necessarily represent those of their affiliated organizations, or those of the publisher, the editors and the reviewers. Any product that may be evaluated in this article, or claim that may be made by its manufacturer, is not guaranteed or endorsed by the publisher.
